# Supplementary material for: Changes in context, typology and programme outcomes between early and recent periods of sex work among young female sex workers in Mombasa, Kenya: A cross-sectional study
Source: PLoS One. 2023 Jul 25;18(7):e0288717. doi: 10.1371/journal.pone.0288717 (PMC10368250; doi:10.1371/journal.pone.0288717)
Supplement: S1 Table — (DOCX) [file pone.0288717.s001.docx]

S1 Table. List of questions

| Variable | Questions |
| --- | --- |
| **Table 1** | |
| Age | Q202, How old were you on your last birth day? Age of each respondent was captured in completed years as on the last birth day. |
| Education Level | Q205, What is the highest level that you completed in school? Level of education was elicited in 9 categories, starting with Never Received Formal Education, Attended Primary but not completed to Completed College and Completed University. |
| Income | Q207, Do you currently have a regular source of income? The responses were Yes and No. |
| Age at first sex | Q304, How many years ago did you first have vaginal or anal sex? Using the number of years ago had first vaginal or anal sex (Q304) and current age (Q202), we computed the age at first sex. |
| Duration in Sex work | Q337, For how long have/had you been in sex work?  Duration since they are in sex work is captured in years (if more than 1 year), in months (if less than 1 year, but more than a week) and in days (if less than a week). We used these three information to compute the duration of sex work, and presented the duration in sex work in years. |
| **Table 2** | |
| Management of sex work | Q342, In the FIRST MONTH OF SEX WORK and the LAST MONTH OF SEX WORK, did you have a pimp, a manager/madam or did you work by yourself?  The options were Had a Pimp, Had a Manager, Worked Self. |
| Number of paying clients per week | Q349, In the FIRST MONTH OF SEX WORK, how many PAYING CLIENTS did you have in a TYPICAL WEEK?  Q357, In the LAST WEEK OF SEX WORK, how many paying clients did you have with? |
| Meeting living expenses through income of sex work | Q335, In the FIRST MONTH OF SEX WORK and the LAST MONTH OF SEX WORK, did the money from your paying clients cover your living expenses in that month?  This question also had the options, None of the expenses, Less than Half of living expenses, Half, More than half and All. |
| Experienced coercion in the past month | q619: In the FIRST MONTH OF SEX WORK, how many men forced you to have sex with them when you were not willing.  We computed if NO men forced to have sex with them, then ‘Not experienced coercion in the first month’, otherwise ‘Yes, experience coercion first month.  q617: In the LAST MONTH, how many men forced you to have sex with them when you were not willing.  We computed if no men forced to have sex with them, then ‘Not experienced coercion in the last month’, otherwise ‘Yes, experience coercion in the last month. |
| Experienced police violence in the past month | Q626, In the FIRST MONTH OF SEX WORK and the LAST MONTH OF SEX WORK, how many times did law enforcement?  The question captured both physical assault and Arrest while on sex work. If they experienced either physical assault or arrest, the study computed as experienced police violence. |
| **Table 3** | |
| Typology of sex work | Q339, How did you meet your PAYING CLIENTS in the FIRST MONTH OF SEX WORK and in the LAST MONTH OF SEX WORK?  Each respondent was asked the different places they meet their clients and a total of 16 different typologies were captures, including ‘others’ if they use other than 15 defined typologies. |
| **Table 4** | |
| Primary Typology | Q340, Of all the different places and ways you had met PAYING CLIENTS, which was the most common for the FIRST MONTH OF SEX WORK and the LAST MONTH OF SEX WORK.  The respondents were asked the most common typology among the listed typologies in Q339. |
| Management of sex work | Q342, In the FIRST MONTH OF SEX WORK and the LAST MONTH OF SEX WORK, did you have a pimp, a manager/madam or did you work by yourself?  The options were Had a Pimp, Had a Manager, Worked Self. |
| Meeting living expenses through income of sex work | Q335, In the FIRST MONTH OF SEX WORK and the LAST MONTH OF SEX WORK, did the money from your paying clients cover your living expenses in that month?  This question also had the options, None of the expenses, Less than Half of living expenses, Half, More than half and All. |
| Number of paying clients per week | Q349, In the FIRST MONTH OF SEX WORK, how many PAYING CLIENTS did you have in a TYPICAL WEEK?  Q357, In the LAST WEEK OF SEX WORK, how many paying clients did you have with? |
| Experienced physical violence from clients in the past month | q607: In the FIRST MONTH OF SEX WORK, of the men with whom you had sex, how many of them physically hurt you? We computed if no men physically hurt, then ‘Not experienced physical violence” and if experienced physical violence from any one partner, then computed as “Experienced physical violence”.  q605: In the LAST MONTH, of the men with whom you had sex, how many of them physically hurt you? We computed if no men physically hurt, then ‘Not experienced physical violence” and if experienced physical violence from at least one partner, then computed as “Experienced physical violence”. |
| Experienced coercion in the past month | q619: In the FIRST MONTH OF SEX WORK, how many men forced you to have sex with them when you were not willing.  We computed if NO men forced to have sex with them, then ‘Not experienced coercion in the first month’, otherwise ‘Yes, experience coercion first month.  q617: In the LAST MONTH, how many men forced you to have sex with them when you were not willing.  We computed if no men forced to have sex with them, then ‘Not experienced coercion in the last month’, otherwise ‘Yes, experience coercion in the last month. |
| Experienced police violence in the past month | Q626, In the FIRST MONTH OF SEX WORK and the LAST MONTH OF SEX WORK, how many times did law enforcement?  The question captured both physical assault and Arrest while on sex work. If they experienced either physical assault or arrest, the study computed as experienced police violence. |
| Living Condition | Q338a-f: The question asked “Thinking back to you when you took your first client, were you, married/divorced/separated/widowed (a-c), Left Home (d), Living with Parents (e), Others (f) [multiple options]. Respondent reported as left home (option **d**), is coded as “Left home”, otherwise, “Living with parents/others”. |
| Age at start of sex work | Q326, How old were you when you took your first paying client with whom the price of sex was negotiated before the sex event? |
| **Table 5** | |
| Ever contacted by a peer/ staff from NGO/ CBO/ FBO | Q803, How many years ago were you first contacted by peers/staff from an NGO, CBO or FBO?  The options were, Number of Months Ago, Number of Years Ago, and Never Been Contacted by Peer/Staff. |
| Ever tested for HIV | Q817, When was the LAST TIME you got tested for HIV? The responses captured were NUMBER OF DAYS AGO, Number of Months Ago and Number of Years Ago. In addition, the response of I HAVE NEVER BEEN TESTED FOR HIV. |
| Ever used a clinic run by BFO/ CBO/ FBO | Q809, Have you ever used any clinics run by an NGO, CBO or FBO? Those responded YES were considered as those ever visited a clinic. |
| Prevalent HIV infection | For each participants, blood samples were collected and tested for HIV infection. The study conducted a first test and a confirmatory test and those found positive in both first and confirmatory tests were treated as positive for HIV infection. |
