# Supplementary material for: Changes in context, typology and programme outcomes between early and recent periods of sex work among young female sex workers in Mombasa, Kenya: A cross-sectional study
Source: PLoS One. 2023 Jul 25;18(7):e0288717. doi: 10.1371/journal.pone.0288717 (PMC10368250; doi:10.1371/journal.pone.0288717)
Supplement: S2 Table — (DOCX) [file pone.0288717.s002.docx]

S2 Table. Change in primary typology of sex work by duration of sex work among young female sex workers in Mombasa, Kenya, 2015

| Duration in sex work | Total | Change in primary typology | |
| --- | --- | --- | --- |
|  |  | Yes (%) | No (%) |
| Total | 403* | 37.2 | 62.8 |
| <6 months | 57 | 45.6 | 54.4 |
| 6-11 months | 40 | 22.5 | 77.5 |
| 1 year | 90 | 33.3 | 66.7 |
| 2 years | 75 | 40.0 | 60.0 |
| 3 years | 62 | 43.5 | 56.5 |
| 4+ years | 79 | 35.4 | 64.6 |

*There were 5 no responses to this question
