# Supplementary material for: Changes in context, typology and programme outcomes between early and recent periods of sex work among young female sex workers in Mombasa, Kenya: A cross-sectional study
Source: PLoS One. 2023 Jul 25;18(7):e0288717. doi: 10.1371/journal.pone.0288717 (PMC10368250; doi:10.1371/journal.pone.0288717)
Supplement: S1 File — (DOCX) [file pone.0288717.s003.docx]

S1 File. Inclusivity in global research

PLOS’ policy on inclusivity in global research aims to improve transparency in the reporting of research performed outside of researchers’ own country or community and ensures that PLOS publications reporting global research adhere to high standards for research ethics and authorship. Authors of relevant research articles may be asked to complete the questionnaire below, which outlines ethical, cultural, and scientific considerations specific to inclusivity in global research. This questionnaire may be requested when researchers have travelled to a different country to conduct research, if research uses samples collected in another country, research with Indigenous populations or their lands, or if research is on cultural artefacts. Researchers travelling to another country solely to use laboratory equipment will not normally be required to complete the questionnaire. However, the questionnaire can be requested at the journal’s discretion for any submission – if you have been requested to complete this questionnaire by the PLOS journal you submitted to, please do so.

Please complete the questionnaire below and include this as a Supporting Information file with your manuscript. Note that if your paper is accepted for publication, this checklist will be published with your article in the supporting information files. Please ensure that you reference the checklist in the main body of your manuscript. We suggest adding a subsection ‘Inclusivity in global research’ to your Methods section and adding the following sentence: “Additional information regarding the ethical, cultural, and scientific considerations specific to inclusivity in global research is included in the Supporting Information (SX Checklist)”

The questions have been designed to be applicable to a wide range of study types, and there are subsections for both human subjects research and non-human subjects research. If any of the questions are not relevant to your research please mark them as “N/A” as appropriate.

**Ethical considerations, permits and authorship**

*This section is applicable to all research types.*

Provide details as to who granted permissions and/or consent for the study to take place in the Methods section of your manuscript. This should include the names of **all** ethics boards, governmental organizations, community leaders or other bodies that provided approval for the study. If individuals provided approval refer to these people by their role or title but do not list their name(s).

The study received ethics approval from the Human Research Ethics Board at the University of Manitoba, Canada (HS16557) and the Kenyatta National Hospital-University of Nairobi Ethical Review Committee, Kenya (P497/10/2017).

Reported on page number: 11

If there were any deviations from the study protocol after approval was obtained please provide details of these changes in the Methods section of your manuscript.
Did this study involve local collaborators that are residents of the country where the research was conducted or members of the community studied? If you do not have any authors from said communities, please provide an explanation for this below.

There were no deviations from the study protocol after approval was obtained.

Reported on page number: N/A

Everyone listed as an author should meet PLOS’ criteria for authorship and all individuals who meet these criteria should be included in the author byline, rather than the acknowledgements. Authorship criteria is based on the International Committee of Medical Journal Editors (ICMJE) Uniform Requirements for Manuscripts Submitted to Biomedical Journals - for further information please see here: <https://journals.plos.org/plosone/s/authorship>.

**Human subjects research (e.g. health research, medical research, cross-cultural psychology)**

Did you obtain written informed consent from a representative of the local community or region before the research took place? How did you establish who speaks for the community? Details of written informed consent obtained from study participants should be reported separately in the Methods section of your manuscript.

In this study we collaborated with a Kenyan organization named International Centre for Reproductive Health – Kenya (ICRH- K). This organization has been working with adolescent girls and women including female sex workers in Mombasa County for several years. Before the study, we had discussions with leadership of the organization. We also had discussion with the FSW peer leaderships who represent the sex workers community. These peer leaders supported us in mapping the hotspots where the sex workers solicit clients and helped us mobilise respondents from the hotspots based on the eligibility criteria.

Our other collaborator was the Ministry of Health through the National AIDS and STI Control Proramme (NASCOP) and the National AIDS Control Council (NACC). These institutions are national bodies involved in developing policy, coordination and management of the national HIV response including the key population programme. Several meetings were organized with these institutions at design stage to understand the key questions that they are struggling to answer. As institutions responsible for the national HIV response, the guided us

How did members of the local community provide input on the aims of the research investigation, its methodology, and its anticipated outcome(s)?

The research study was designed to respond to the concern shared by the female sex workers community and ICRH that the HIV prevention programme may have been reaching the sex workers late in their lives. There was a strong feeling that the programmes reach the sex workers few years after they initiate sex work. This gap between entry into sex work and reach by the programme may increase risk and vulnerability to HIV for young sex workers. Based on this concern the aim of the research was decided as follows:

**Objective 1**: To describe how the characteristics and length of the transition period and access gap varies across three epidemiological contexts (Bangalore, India; Nairobi, Kenya and Zaporizhzhya, Ukraine).

**Objective 2**: To understand how the risk of HIV varies by length and characteristics of the transition period and access gap across three epidemiologic contexts.
**Objective 3:** To assess the extent to which HIV infections acquired during the transition period and access gap (before women are reached by existing TIs), could mitigate the population level impact of TIs for self-identified FSWs, and to explore the potential marginal benefit of expanding programs to reach women during the transition period and access gap, across epidemiologic contexts.

When engaging with the local community, how did you ensure that the informed consent documents and other materials could be understood by local stakeholders?

The study design and protocol were shared with the peer leaders in ICRH – K and a 5 days training was conducted with the leaders to explain the study design, eligibility criteria, sampling, consent documents, questionnaire etc. Several role-plays and practice sessions were done during the training to confirm that the peer leaders understood the study well and would be able to communicate to their peers. The peer leaders described the study to their peers at the hotspot so that the community is aware of the study and its objectives.

Will the findings of the research be made available in an understandable format to stakeholders in the community where the study was conducted (e.g. via a presentation, summary report, copies of publications, etc.)? Please provide details of how this will be achieved.

The findings of the research were shared with the peer educators working in ICRH-K twice and several times with the National AIDS Control Council (NACC) and National AIDS and STI Control Programme (NASCOP). A special dissemination workshop was organized to disseminate the findings. The findings were presented in the national key population Technical Working Group and other webinars organized by NACC. The findings have been also presented in several conferences as posters and oral presentations and more than 10 papers have been published disseminating the results of the study.

**Non-human subjects research using specimens/ animals collected as part of the study, or those housed in archival collections. Examples include archaeology, paleontology, botany and zoology.**

Did the permission you obtained from a local authority to perform the study include an agreement on access to outputs and benefit sharing? This may include procedures to enable fair distribution of the benefits and resources arising from the research performed. Please include any details of Prior Informed Consent and Benefit Sharing Agreements obtained. These may be required by field-specific regulations, for example the Convention on Biological Diversity (CBD) and the associated Nagoya Protocol.

NA

If the material used in your study was imported, please A) provide the year it was imported and B) indicate whether permits were obtained to import/export the materials used, C) provide details of any permits obtained. If this information is not available, please indicate this.

No

If you used archival specimens, please state how the material used in your study was acquired by the institute it is held in and provide details of any permits obtained for the original excavations/ sample collection. If this information is not available, please indicate this.

No

How was the potential cultural significance of the materials collected in your study to local communities considered in your research design? Were Indigenous peoples and/or local researchers and institutions involved with archaeological excavations / collection of specimens? If so, please provide a description of their involvement.

NA

If your manuscript includes photographs of human remains please indicate whether authors obtained permission from descendants or affiliated cultural communities to do so.

NA
