# Supplementary material for: Changes in context, typology and programme outcomes between early and recent periods of sex work among young female sex workers in Mombasa, Kenya: A cross-sectional study
Source: PLoS One. 2023 Jul 25;18(7):e0288717. doi: 10.1371/journal.pone.0288717 (PMC10368250; doi:10.1371/journal.pone.0288717)
Supplement: Attachment — Submitted filename: PLOSONE.docx [file pone.0288717.s004.docx]

In the manuscript *“Variability in the context between early and recent periods of sex work among young female sex workers in Mombasa, Kenya: a cross-sectional study,”* authors use data from a 2015 cross-sectional survey with 408 female sex workers to assess differences in context and practices of sex work between the first month of the woman’s engagement in sex work and more recent sex work because differences may implicate a need for different time-period tailored HIV prevention programs. While the research question is important and study has potential for high impact, it has several shortcomings which need to be addressed.

Abstract: Consider briefly defining ‘typology’ and ‘early’ vs. ‘recent’ as these terms are not readily apparent/familiar to readers who do not commonly conduct research/programs with this population. The Results statement beginning with “higher proportion among street/bus stop typology” seems incomplete and should be reworded for clarity.

Intro: For clarity, consider defining “early” vs “recent” period and age criteria for “YFSW” early on in the intro (i.e., at first reference). Also, consider expanding on the hypothesis that HIV/STI risk may vary by change in typology and sex work practices over time. Why and how might change in typology and sex practices change HIV risk? Why/how this could impact HIV prevention strategies? This will further justify research question and need for the study.

Methods:

- As a key aim of the study is to assess change in typology of sex work over time, how adequate were current recruitment methods in comprehensively identifying/recruiting participants across dimensions of typology? i.e., if participants were recruited from sex work venues, would participant who engaged in home-based or internet-based sex work be adequately identified? The period of “early” vs. “recent” could have different impact based on how long the participant had been involved with sex work. For example, we might expect little variation in someone who had only been employed for 2 months vs 24 months in sex work. How was this handled?
- Were participants reimbursed for participation? What was the length of the survey? How was the privacy and safety of the participants ensured?
- More details regarding how the predictors “physical violence,” “experience of coercion,” and “living condition” were assessed and analyzed is needed.
- The study outcomes should be identified and defined consistently. For example, lines 151-152 describe planned assessment of the impact of primary typology on “HIV prevalence” and “program reach.” Is prevalence the right outcome? Perhaps HIV status (negative vs. positive) is more appropriate? How is program reach defined and measured? The Statistical Analysis section only discusses testing the association between change in primary typology and factors associated with change in typology. This is discrepant from the prior section.

Results:

- Table 1 should depict the spread of values for individuals engaged in sex work <2 years.
- Table 2 should include physical violence and living condition as described in the Methods
- Table 3 text should describe overlap between typologies. How is this handled/depicted in the table?
- A key is needed to explain shading in Figure 1
- Table 4 and 5 don’t seem to be hypothesis driven or aligned with the paper. For example, the analysis presented answers what factors drove change in typology and impact of change in typology on outcome. I think the real question that authors are interested in is the switch from a lower-risk to a higher-risk typology and this is not being assessed in the current analysis.

Overall comments: The manuscript could benefit from grammar/spelling proofing.
